# The Methods of Using Gold and Tin Together

**Published:** 1898-04

**Authors:** C. J. Lyons


### The Methods of Using Gold and Tin Together.

BY C. J. LYONS.

In treating this subject I shall divide it into two parts, viz.: the method of using tin and gold together, making a combination known as tin-gold, and the method of using tin or tin-gold, with pure gold as a capping.

The material of gold and tin known as tin-gold is prepared by laying a sheet of No. 4, 5 or 6 non-cohesive gold foil upon a sheet of No. 4 extra tough tin foil, cutting them into 2x4 strips, and rolling them together into a soft rope; they may then be cut into short pieces, or they may be used whole, in a manner similar to soft gold ribands. The material is inserted on exactly the same principle as fillings of non-cohesive gold. The instruments are the same as used for non-cohesive gold, except that the chief requisite being they have square and not round points, as a large part of the packing is done with the side of the instrument.

In combinations this way, it makes no difference if a slight amount of moisture gets on the receiving surface during filling, as it does not interfere with the progress or success of the operation, for the material becomes consolidated subsequent to its insertion, owing to an electro-chemical process, through which the tin is dissolved and redeposited upon the surface of the gold, and by this means the material becomes rigid and all parts of the filling thoroughly bound together; at the same time a slight expansion of the material takes place, leading to a more complete closure of the cavity. In tin-gold the wedge principle is used for holding in the filling. One-fourth sheet of tin and three-fourths of a sheet of gold make the best combination, but larger proportions of tin may be used successfully. For large cavities one-half, two-thirds, or even whole sheets, can be rolled and used when cut into cubes or blocks.

To obtain good results with this combination, it must be used with the same care and accuracy that are required for working gold.

Now, as to the method of using tin or tin-gold, with pure gold as a capping.

To secure good results from the use of tin, it is necessary to pack it to a state of considerable density. To do this, however, it requires great dexterity, for, on account of the entire absence of cohesion and extreme softness, the pieces of foil do not keep their places as well as gold. On account of this tendency and of its great softness, it is best, in starting, to use quite large blocks of the foil, wedging them together until a secure foundation is effected, when the remainder may generally be added without difficulty.

This foil does not well bear malleting, excepting for the purpose of effecting surface consolidation, when the instruments should have broad faces. If, however, it is used in conjunction with a matrix, there is no objection to the mallet, since the blocks can not escape from the cavity, and are, therefore, driven into form and thorough adaptation to the walls.

The surface of the filling should be well consolidated before beginning to insert the gold for the gold capping.

In cavities with strong overhanging walls of enamel, which it may not be convenient or desirable to remove, they can be filled to the neck with tin or tin-gold and a wedge-shaped instrument is forced into the center of the filling driving the material against the wall of the cavity, and the opening thus made filled with gold. All very large and deep crown cavities can be filled the same way, the central gold plug forming a key to the filling. It may be very small or it may be extended quite to the margin of the cavity, so that when the operation is completed only the gold will be seen.

In filling compound proximal cavities with tin and gold, the matrix can be employed to advantage. A pellet of the tin or tin-gold is pressed against the buccal wall, a second against the lingual, a third as a wedge between the two, and all driven against the cervical wall with a mallet. This operation is repeated until one-third to three-fourths of the proximal part of the cavity is filled with tin or the tin-gold, then begin the gold on the grinding surface and build it over the tin-gold. Where the grinding surface is not involved, we obtain retention for the gold cap in short grooves in the lingual or buccal wall.

